# RU486 did not exacerbate cytokine release in mice challenged with LPS nor in db/db mice

**DOI:** 10.1186/1471-2210-8-7

**Published:** 2008-05-12

**Authors:** Baichun Yang, Ryan P Trump, Ying Shen, Judi A McNulty, Lisa G Clifton, Stephen A Stimpson, Peiyuan Lin, Greg L Pahel

**Affiliations:** 1 Department of Metabolic Molecular Pharmacology, Research & Development, GlaxoSmithKline, Research Triangle Park, USA; 2 Department of Discovery Molecular Chemistry, Research & Development, GlaxoSmithKline, Research Triangle Park, USA

## Abstract

**Background:**

Glucocorticoids down-regulate cytokine synthesis and suppress inflammatory responses. The glucocorticoid receptor (GR) antagonist RU486 may exacerbate the inflammatory response, and concerns over this exacerbation have limited the development and clinical use of GR antagonists in the treatment of diabetes and depression. We investigated the effects of RU486 on serum cytokines in db/db mice and on lipopolysaccharide (LPS)-induced circulating TNFα levels in both normal AKR mice and diet-induced obese (DIO) C57BL/6 mice.

**Results:**

Chronic treatment of db/db mice with RU486 dose-dependently decreased blood glucose, increased serum corticosterone and ACTH, but did not affect serum MCP-1 and IL-6 levels. LPS dose-dependently increased serum TNFα in both AKR and C57BL/6 DIO mice, along with increased circulating corticosterone and ACTH. Pretreatment of the mice with RU486 dose-dependently suppressed the LPS induced increases in serum TNFα and further increased serum corticosterone.

**Conclusion:**

RU486 at doses that were efficacious in lowering blood glucose did not exacerbate cytokine release in these three mouse models. RU486 actually suppressed the lower dose LPS-mediated TNFα release, possibly due to the increased release of glucocorticoids.

## Background

Various clinical and pre-clinical investigations have indicated that antagonists of the glucocorticoid receptor (GR) could be useful in the treatment of diabetes [1,2] and depression [3], but concerns about the effects of GR antagonists on the body's ability to regulate inflammatory responses [4] have hampered development of GR antagonists for these indications.

Activation of the GR with endogenous glucocorticoids (GCs) is the body's primary method for suppression of the inflammatory response [5]. In fact, host survival in bacterial and viral infection is dependent upon the proper control of the inflammatory response through timely activation of the hypothalamic-pituitary-adrenal (HPA) axis for the production of cortisol, the primary glucocorticoid in humans [6]. The innate immune system stimulates the controlled production and timely release of GCs to prevent an overly strong response to an ongoing localized inflammatory process [7]. Disruption of this response due to exhaustion of the adrenal cortex results in septic shock. In the treatment of septic shock, low doses of GCs have therapeutic effects by correcting adrenal cortex exhaustion, exerting appropriate anti-inflammatory properties, and enhancing endogenous catecholamine effects [8].

Antagonism of the GR by mifepristone (RU486), pharmacologically classified as both a progesterone and glucocorticoid antagonist [9], has been shown to ameliorate metabolic parameters in rodent model of type 2 diabetes (T2D) [10]. However, the potential for GR antagonists to exacerbate inflammation is a major concern limiting the use of GR antagonists for the treatment of diabetes, depression, and other conditions. It was postulated that RU486 could exacerbate the inflammatory response and lead to septic shock through inhibition of the body's mechanism for controlling inflammation by virtue of its ability to block the GR in phagocytes located at the site of invading bacteria [11], by disrupting the negative pituitary feedback, and by deteriorating adrenal cortex exhaustion [12]. In experimental animals, blockade of the GR by RU486 was shown to increase the mortality of endotoxemic rats administered lipopolysaccharide, and to increase TNFα production and toxicity [13]. There have also been reports of deaths from septic shock in patients using RU486 as an abortifacient [14-16].

The association between use of RU486 and deaths from septic shock has only been reported under the condition of abortion (via progesterone receptor antagonism). No causal relationship has been established between RU486 and septic shock. The use of RU486 in Cushing's syndrome [17-19], breast cancer [20], endometriosis [21], and leiomyoma [22] has not been associated with septic shock, although all the applications are also based on either progesterone receptor antagonism or GR antagonism. The incidence of septic shock may depend on disease conditions and RU486 dosages.

In the effort to develop GR antagonists for chronic treatment of diabetes, we desired to investigate the acute and chronic effect of GR antagonist on the inflammatory responses in animals under both normal and disease conditions at doses that are efficacious in the treatment of T2D. Therefore, the current study investigated the effect of acute and chronic use of RU486 on inflammatory cytokines in various mouse models, including normal AKR mice and two rodent models of T2D, the monogenic leptin receptor defect db/db mice and the diet-induced obese (DIO) C57BL/6 mice.

## Results

### RU486 dose-dependently decreased blood glucose but did not affect serum cytokines in db/db mice

**Figure 1:**
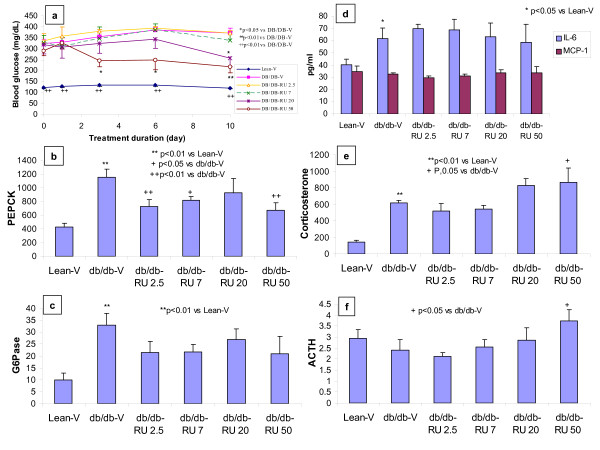
**Effects of RU486 (oral, BID, 10 days) on blood glucose (a), liver genes of PEPCK (b) and G6Pase (c), and serum IL-6 and MCP-1 (d), corticosterone (e), and ACTH (f) in db/db mice.** Lean-V: lean litter mates treated with vehicle. db/db-V: db/db mice treated with Vehicle. db/db-RU 2.5: db/db mice treated with RU486 2.5 mg/kg. db/db-RU 7: db/db mice treated with RU486 7 mg/kg. db/db-RU 20: db/db mice treated with RU486 20 mg/kg. db/db-RU 50: db/db mice treated with RU486 50 mg/kg. Serum corticosterone and ACTH were presented as ng/ml. PEPCK and G6Pase were normalized with cyclophilin.

At an age of 11–12 weeks, db/db mice had significantly higher levels of blood glucose, HbA1c, serum insulin, total cholesterol, triglycerides, and interleukin-6 (IL-6), and similar level of serum monocyte chemoattractant protein-1 (MCP-1) compared with the C57BL/6J lean litter mates (Fig 1 and Table 1). Serum tumor necrosis factor alpha (TNFα) and interleukin-1beta (IL-1β) were below quantifiable levels (BQL) in both db/db mice and their lean litter mates. The db/db mice also had significantly higher levels of serum corticosterone, similar levels of adrenocorticotropic hormone (ACTH), and higher expression levels of hepatic glucogenesis enzyme phosphenolpyruvate carboxykinase (PEPCK) and glucose-6-phosphatase (G6Pase) genes compared with the C57BL/6J lean litter mates (Fig 1 and Table 1).

**Table 1:** Phenotype of db/db mice

	Lean litter mates	db/db mice
Body weight (g)	24.1 ± 0.6	39.8 ± 0.5**
Blood		
Glucose (mg/dL)	120.1 ± 4.6	319.9 ± 37.5**
HbA1c (%)	3.2 ± 0.2	5.4 ± 0.6**
Serum		
Insulin (ng/ml)	0.3 ± 0.1	12.6 ± 2.9**
Total cholesterol (mg/dL)	73.8 ± 1.8	127 ± 3.2**
Triglycerides (mg/dL)	125.4 ± 15.8	200.9 ± 19.5**
IL-6 (pg/ml)	40.1 ± 4.5	61.8 ± 8.6*
MCP-1 (pg/ml)	34.4 ± 5.0	32.4 ± 1.0
TNFα	BQL	BQL
IL-1β	BQL	BQL
Corticosterone (ng/ml)	45.7 ± 17.8	615 ± 36.4**
ACTH (ng/ml)	2.9 ± 0.4	2.4 ± 0.5

* p < 0.05 vs lean litter mates. **p < 0.01 vs lean litter mates.

BQL: Below quantifiable level.

Treatment of the db/db mice at an age 10–12 weeks with GR antagonist RU486 for 10 days significantly decreased postprandial blood glucose in a dose- and time-dependent manner (Fig 1a), confirming the glucose lowering effect of RU486 and establishing the efficacious dose. RU486 down-regulated hepatic PEPCK expression levels (Fig 1b), and trended to lower G6Pase (Fig 1c) gene expression, and increased serum levels of corticosterone (Fig 1e) and ACTH (Fig 1f) in db/db mice. RU486 did not affect serum MCP-1 and IL-6 levels in the db/db mice (Fig 1d).

### RU486 decreased LPS-induced serum TNFα in normal AKR mice along with an increase in serum corticosterone

**Figure 2:**
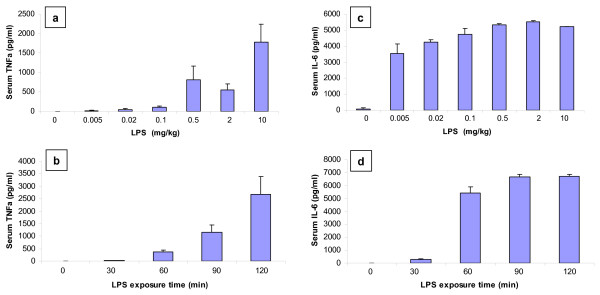
**LPS induced dose-dependent TNFα (a) and IL-6 (c) production and LPS induced time-dependent TNFα (b) and IL-6 (d) production in AKR mice.** LPS exposure time was 90 minutes in (a) and (c). LPS dose in (b) and (d) was 0.5 mg/kg.

As shown in Fig 2, ninety minutes after LPS intraperitoneal (ip) injection, serum TNFα and IL-6 levels were increased in a dose- and time-dependent manner. The LPS doses of 0.1 and 0.5 mg/kg were the at the lower part of the dose-response curve for TNFα induction (Fig 2a), indicating that these doses should be far from the exhausting capacity for TNFα production. The 90 minute exposure time was also in the middle of TNFα release curve (Fig 2b). The LPS-induced IL-6 production, which was much more sensitive than TNFα production, reached a maximal level at an LPS dose of 0.5 mg/kg 90 minutes after exposure (Fig 2c and Fig 2d). In the subsequent studies of LPS challenge in AKR mice, the middle LPS doses of 0.1 and 0.5 mg/kg, an exposure time of 90 minutes, and the parameter of TNFα production were employed.

**Figure 3:**
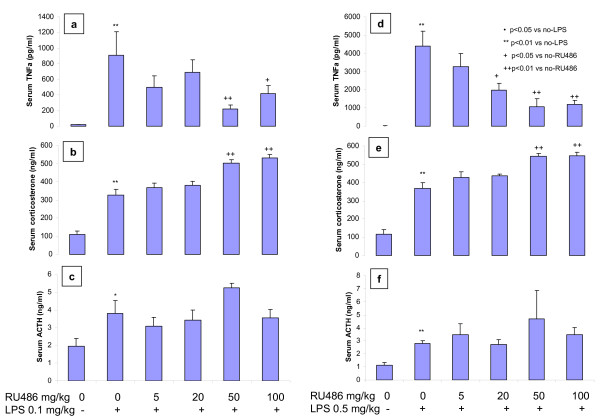
**Effects of acute RU486 treatment on LPS (0.1 and 0.5 mg/kg)-induced changes in serum TNFα (a and d), corticosterone (b and e), and ACTH (c and f) in normal AKR mice.** RU486 was oral gavaged 30 minutes prior to ip LPS. LPS exposure time was 90 minutes.

While LPS doses of 0.1 and 0.5 mg/kg increased serum TNFα to different levels, oral RU486 significantly inhibited both LPS 0.1 and 0.5 mg/kg-induced increase in serum TNFα. This inhibition trended to be dose-dependent and is opposite to the proposed pro-inflammation effects of RU486 (Fig 3a and Fig 3d). Both LPS 0.1 and 0.5 mg/kg resulted in significantly higher serum corticosterone (Fig 3b and Fig 3e) and ACTH (Fig 3c and 3f). Pre-treatment with RU486 further increased serum corticosterone in these animals, but did not affect the LPS-elevated ACTH (Fig 3c and Fig 3f).

### RU486 decreased low dose LPS-induced serum TNFα in C57BL/6 DIO mice along with an increase in serum corticosterone, but did not affect the high dose LPS-mediated changes

**Figure 4:**
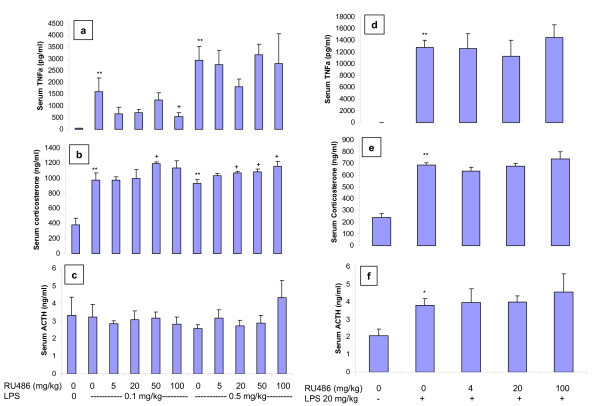
**Effects of acute RU486 treatment on lower doses of LPS (0.1 and 0.5 mg/kg)-induced changes in serum TNFα (a), corticosterone (b), and ACTH (c), and on high dose LPS (20 mg/kg)-induced changes in serum TNFα (d), corticosterone (e), and ACTH (f) in C57BL/6 DIO mice.** RU486 was oral gavaged 30 minutes prior to ip LPS. LPS exposure time was 90 minutes. * p < 0.05 vs no-LPS. ** p < 0.01 vs no-LPS. + p < 0.05 vs no-RU486.

After 12–13 weeks on cafeteria diet, C57BL/6 mice had a phenotype of higher body weight, serum glucose and insulin, dyslipidemia, and had higher serum TNFα and similar IL-6 (Table 2) compared to normal diet controls. Acute LPS challenge in C57BL/6 DIO mice markedly increased serum TNFα in a dose-dependent manner (Fig 4), which was more pronounced than that in normal diet controls (with LPS 20 mg/kg, 12852 ± 1185 pg/ml vs 6494 ± 1655 pg/ml in normal diet controls, p < 0.05). Pretreatment with RU486 decreased the low dose 0.1 mg/kg LPS-induced serum TNFα, but did not affect the higher dose 0.5 mg/kg LPS-elevated serum TNFα (Fig 4a). LPS increased serum corticosterone in the C57BL/6 DIO mice (Fig 4b). Pretreatment with RU486 further increased the LPS (0.1 and 0.5 mg/kg)-elevated serum corticosterone but did not affect serum ACTH (Fig 4b and 4c).

**Table 2:** Phenotype of C57BL/6 DIO mice

	Normal diet controls	DIO mice
Body weight (g)	28.7 ± 0.3	44.6 ± 0.6**
Blood		
Glucose (mg/dL)	121 ± 3	160 ± 6*
HbA1c (%)	3.6 ± 0.2	3.5 ± 0.3
Serum		
Glucose (mg/dL)	225 ± 15**	344 ± 22*
Insulin (ng/ml)	0.45 ± 0.06	5.07 ± 1.03**
Total cholesterol (mg/dL)	67.0 ± 3.3	223.0 ± 3.1**
Triglycerides (mg/dL)	93.0 ± 14.6	138.0 ± 9.8*
FFA (mEq/L)	0.35 ± 0.03	0.47 ± 0.02*
IL-6 (pg/ml)	191.2 ± 86.7	243.2 ± 144.7
TNFα(pg/ml)	24.0 ± 1.1	35.6 ± 2.4*
Corticosterone (ng/ml)	310 ± 30	380 ± 86
ACTH (ng/ml)	2.3 ± 0.2	3.3 ± 1.0

* p < 0.05 vs lean litter mates. **p < 0.01 vs lean littermates.

In another experiment, C57BL/6 DIO mice were ip injected with LPS at the dose of 20 mg/kg. This high dose of LPS led to inactivity and diarrhea in mice, an exaggerated increase in serum TNFα, and moderate increases in serum corticosterone and ACTH (Fig 4d–f). Pretreatment with RU486 in the C57BL/6 DIO mice did not affect the high dose LPS-mediated changes in serum TNFα, corticosterone, and ACTH (Fig 4d–f).

## Discussion

The current study demonstrated that chronic treatment with GR antagonist RU486 for 10 days dose-dependently decreased postprandial blood glucose, increased serum corticosterone and ACTH, and did not affect serum MCP-1 and IL-6, in db/db mice. Injection of LPS increased circulating TNFα, corticosterone, and ACTH in both normal AKR mice and C57BL/6 DIO mice. RU486 suppressed the lower dose LPS-elevated serum TNFα in both normal AKR mice and C57BL/6 DIO mice, and further increased circulating corticosterone. The RU486 pretreatment did not affect the high dose LPS (20 mg/kg)-mediated changes in serum TNFα and corticosterone. It is worth noting that the data sets from each strain/model examined were not exactly the same in current studies. But all the studies showed that RU486 either decreased cytokine along with increased corticosterone, or did not affect cytokine along with or without change in corticosterone. Thus RU486, at doses which are efficacious on glucose lowering, did not show any exacerbating effect on cytokine production in both chronic and acute settings, and RU486 could actually suppress the lower dose LPS-mediated TNFα release, possibly due to the increased release of GCs. The interpretation of the data sets were discussed below.

Although GR antagonism has been shown to inhibit gluconeogenesis and lower glucose in humans [1] and in rodent model of T2D [23], the concern about GR antagonists on pro-inflammation hampers the long-term treatment with GR antagonists in T2D. The current study used db/db mice to determine the effect of chronic RU486 on circulating cytokines, which is a simulative study to mimic drug use in patients with T2D. The authors found that RU486 dose-dependently decreased postprandial blood glucose, increased serum corticosterone and ACTH, but did not affect serum MCP-1 and IL-6, although the basal IL-6 was higher in db/db mice than in the lean littermate controls. The significantly higher level of IL-6 in the db/db mice is consistent with a state of chronic low-grade inflammation. The changes in glucose, corticosterone, and ACTH in RU486-treated db/db mice were consistent with relevant literature reports, and confirmed the efficacious drug exposure (measured serum drug level 2–3 hours after last dose: 14 ± 3, 33 ± 3, 273 ± 17, and 719 ± 57 nM, respectively at doses of 2.5, 7, 20, and 50 mg/kg). These findings suggest that RU486 did not further enhance inflammation in this mouse model of T2D with low grade inflammation. The dose-effect curves of RU486 on glucose, corticosterone, and ACTH were used to decide the RU486 doses 4–100 mg/kg used in the subsequent acute studies.

In the treatment of septic shock, low doses of glucocorticoids have anti-inflammatory properties. GR antagonists might block the anti-inflammatory effect of endogenous cortisol, and deteriorate inflammation. Excessive LPS release from the bacterial wall into the circulation is the primary pathogenic process of septic shock [24]. To evaluate the effect of RU486 under the condition of septic shock, an LPS-challenge model was used in the current studies. We found that in normal AKR mice, LPS dose-dependently increased serum TNFα and caused increases in corticosterone and ACTH. Since LPS-increased circulating TNFα and other circulating cytokines can trigger the neural circuits that control the HPA axis [25], the increases in corticosterone and ACTH in response to LPS challenge were an expected stress response of HPA axis [26]. Interestingly, pretreatment with RU486 dose-dependently decreased serum TNFα, which is contrary to the hypothesis that RU486 would exacerbate inflammation. There was also a dose-dependent increase in circulating corticosterone in response to RU486 treatment, which is a well known compensatory reaction in response to the GR antagonism. Thus the suppression of LPS-mediated TNFα production in RU486-treated mice was most likely a consequence of the increased circulating corticosterone. Because of the compensatory increase in endogenous glucocorticoids, acute GR antagonism with RU486 does not necessarily lead to exacerbation of inflammation.

It has been reported that diet-induced obesity or/and diet-induced insulin resistance in mice is associated with low grade inflammation, as evidenced by macrophage accumulation in adipose tissue and increased serum cytokines [27,28]. Chronic low-grade inflammation in these mice could alter the effects of RU486 on LPS-induced TNFα production compared to the effects in normal mice. Therefore, we used C57BL/6 DIO mice to determine the effects of LPS and RU486. The phenotype of the C57BL/6 DIO mice was consistent with the literature [29]. While the LPS-induced increases in serum TNFα and corticostrone were similar to those in normal AKR mice, the effects of RU486 pretreatment on the LPS-mediated changes in C57BL/6 DIO mice were different than that in the normal AKR mice. RU486 suppressed 0.1 mg/kg LPS-induced TNFα production but did not affect 0.5 mg/kg LPS-induced TNFα production, despite the fact that RU486 further increased serum corticosterone at both LPS doses. Thus, under these conditions, the compensatory increase in glucocorticoids was not able to suppress LPS-induced TNFα production.

Considering that the stress response during sepsis could exhaust adrenal cortex, a very high dose of exogenous LPS could mimic this state and would exhaust the endogenous glucocorticoids. Under these conditions, RU486 may no longer be able to cause compensatory glucocorticoid release. Whether RU486 pretreatment exacerbates LPS-induced TNFα production under this condition is not known. Therefore, we used LPS 20 mg/kg in C57BL/6 DIO mice to observe the effect of RU486 in this stressed condition. We found that this very high dose of LPS led to an exaggerated increase in serum TNFα, but increases in serum corticosterone and ACTH were similar to those observed after LPS 0.1 and 0.5 mg/kg. RU486 pretreatment did not affect the 20 mg/kg LPS-mediated changes in TNFα, corticosterone, and ACTH. Despite this lack of increase in glucocorticoids, RU486 did not enhance TNFα production.

There are a few papers reporting GR agonist activity of RU486 in some in vitro and in vivo systems. Nordeen et al [30] reported that protein kinase A activators unmasked the agonist effect of RU486 as mediating an induction of hormone-responsive report genes. Zhang et al [31] reported that the GR agonist activities of RU486 were dependent on the GR levels but not on EC50 values in COS-7 cells transfected with GR expression vector. Schulz et al [32] reported that the RU486-induced GR agonism was controlled by the receptor N terminus and by corepressor binding. All these reports indicated that RU486 may act as a GR agonist under certain conditions on certain pathways/procedures. This agonist activity of RU486 may contribute to our observation of decreasing LPS-induced TNFα by RU486 in normal AKR mice and C57Bl/6 DIO mice. Although the mechanism and extent of GR agonist activities of RU486 in whole animals is not known, the GR agonist activities of RU486 reduce the extent of, and concerns about, potential inflammatory exacerbation by the drug.

As demonstrated in the current studies, LPS challenge is a strong stress to the subjects. LPS not only increases cytokine production (such as TNFα and IL-6), but also stimulates HPA axis and leads to increases in both serum corticosterone and ACTH. The levels of cytokine, ACTH, and corticosterone are considered to be the homeostatic status under the LPS-challenge. In the HPA axis, GCs have negative feedback effects on hypothalamus and anterior pituitary gland. GR antagonism with RU486 blocks the GR throughout body tissues and even blocks GCs' negative feedback receptors in the hypothalamus and anterior pituitary gland. This blockade leads to activation of HPA, and results in increased rate of synthesis and release of ACTH and GCs. The current studies in AKR mice and C57BL/6 DIO mice demonstrated that the acute RU486 pretreatment resulted in an increase only in corticosterone but not in ACTH. This finding indicates that GCs may have a negative feedback on the adrenal cortex, thus regulating its own synthesis and release.

Although the 5 deaths of septic shock were reported to be associated with use of RU486 [14-16], and antagonism of the GR was hypothesized to be the primary cause [16], the current studies in three different strains of animals with normal and abnormal metabolism have not shown inflammatory exacerbation by RU486 treatment. This suggests, notwithstanding the species and strain differences, that mechanisms other than, or along with, antagonism of the GR may contribute to the inflammatory exacerbation exhibited by RU486 in obstetric/gynecologic clinic. RU486 also blocks progesterone receptors [33], and progesterone also demonstrates anti-inflammatory properties [34-37]. Therefore, the septic shock associated with use of RU486 as abortifacient may not be solely due to antagonism of the GR by RU486. The exact cause of these deaths needs to be further investigated.

## Conclusion

Our studies demonstrated that chronic treatment with GR antagonist RU486 in db/db mice and acute use of RU486 in LPS-challenged normal and DIO mice did not show any evidence of exacerbating cytokine production. These findings suggest that the compensatory increase in circulating GCs induced by RU486 treatment results in an net anti-inflammatory effect, and suggest that the septic shock associated with use of RU486 as an abortifacient is not solely due to GR antagonism.

## Methods

### Experimental animals

All procedures performed were in compliance with the Animal Welfare Act and U.S. Department of Agriculture regulations, and were approved by the GlaxoSmithKline Animal Care and Use Committee. Male AKR mice (8–9 weeks old) were purchased from Jackson Laboratory (Bar Harbor, ME) and were fed rodent chow Purina 5001 (PMI Nutrition International, LLC, Brentwood, Mo). Db/db mice (8–9 weeks old) and C57BL/6J lean litter mates (for db/db control, 8–9 weeks old) were purchased from Jackson Laboratory (Bar Harbor, ME) and were fed PMI 5K67 diet (PMI Nutrition International, LLC, Brentwood, Mo). Male C57BL/6 mice (5–6 weeks) were purchased from Taconic (Hudson, NY) and were fed cafeteria diet (rodent chow Purina 5001, Lard diet with 60% fat source calorie, and condensed milk diet, Research Diets, Inc., New Brunswick, NJ) for 12–13 weeks (preparing the diet-induced obesity mice, i.e., DIO mice), or fed rodent chow Purina 5001 for normal diet controls. Animals ate and drank *ad libitum*. AKR mice and db/db mice were acclimatized for 1 week before experimental procedures.

### RU486 in db/db mice

Db/db mice were monogenic leptin receptor defect mice, with a phenotype of T2D. This strain of mice was used to simulate the effect of RU486 on T2D subjects.

Six groups of mice (n = 5~9/each group) were assigned as follows: db/db mice treated with RU486 0 (vehicle), 2.5, 7, 20, and 50 mg/kg, BID, PO, and C57BL/6J lean litter mates treated with vehicle 0.5% hydroxypropyl methylcellulose-0.1% tween 80 10 ml/kg, BID, PO, for 10.5 days. Postprandial blood glucose was monitored before starting treatment and 1, 3, 6, and 10 days after treatment between 7–8 am prior to morning dosing, via tail snip (3–4 mm from the tip) using Elite^® ^XL Glucometer (Bayer, Tarrytown, NY). 2–3 hours after the last dose, mice were anesthetized under isoflurane. Blood was collected via cardiac stick for measuring HBA1c and obtaining serum. Liver samples were collected for determining gene expression levels of glucogenesis enzyme PEPCK and G6Pase. Serum was used for measuring inflammatory parameters TNFα, MCP-1, IL-1β, andIL-6; HPA axis parameters ACTH, and corticosterone; and metabolic parameters glucose, insulin, total cholesterol, and triglycerides.

### RU486 in AKR mice acutely challenged with lipopolysaccharide (LPS)

AKR mice, at age 9–10 weeks old and fed ordinary rodent diet, were normal in metabolism [38]. This strain of mice was used to simulate the effect of RU486 on LPS-induced cytokine release in healthy subjects.

To demonstrate the effect of LPS on serum cytokine production, one set of AKR mice was intraperitoneally (ip) injected with LPS 0.001–10 mg/kg (in saline), then subjected to blood collection via cardiac stick under isoflurane 90 minutes after LPS injection; another set of AKR mice was ip injected with LPS 0.5 mg/kg, then subjected to blood collection via cardiac stick under isoflurane 30, 60, 90, and 120 minutes after LPS injection. Serum separated from the blood samples was analyzed for TNFα, IL-6, corticosterone, and ACTH. To determine the effect of RU486 on LPS-stimulated cytokine production *in vivo*, AKR mice were orally gavaged with vehicle or RU486 5, 20, 50, or 100 mg/kg. Thirty minutes later, the mice were ip injected with LPS 0.1 or 0.5 mg/kg. Blood samples were collected via cardiac stick under isoflurane 90 minutes after LPS injection. Serum separated from the blood samples was analyzed for TNFα, corticosterone, and ACTH.

### RU486 in C57BL/6 DIO mice acutely challenged with lipopolysaccharide (LPS)

C57BL/6 DIO mice were mice with abnormal metabolism resulted from over eating and high fat diet. This strain of mice was used to simulate the effect of RU486 on LPS-induced cytokine release in T2D subjects.

To determine the effect of RU486 on LPS-stimulated cytokine production in animals with metabolic disorders, one set of C57BL/6 DIO mice was orally treated with vehicle or RU486 4, 20, 100 mg/kg. Thirty minutes later, the mice were ip injected with LPS 0.1, 0.5 or 20 mg/kg. Blood samples were collected via cardiac stick under isoflurane 90 minutes after LPS injection. Serum separated from the blood samples was analyzed for TNFα, corticosterone, and ACTH.

### Bioassays

Serum TNF-α and IL-6 were measured using ELISA kits (cat. # EMTNFA5 and EM2IL65 respectively) from Pierce Biotechnology, Inc. (Rockford, IL). Serum MCP-1 and IL-1β were measured using a customized MSD kit of Meso Scale Diagnostic, LLC (Gaithersburg, MD). Serum corticosterone was measured using an ELISA kit OCTEIA Corticosterone (Ref. AC-14F1) of IDS Inc. (Fountain Hills, AZ). Serum ACTH was measured using an ELISA kit ACTH 1–39 (cat. # S-1130) of Peninsula Laboratories, Inc (San Carols, CA). Serum glucose, total cholesterol, and triglycerides were measured with the Olympus Au640^® ^clinical chemistry analyzer (Serial #2040881, Olympus America Inc, Diagnostic Systems Group, Two Corporate Center Drive, Melville, NY). Blood HBA1c was measured with the Bio-Rad Variant II Hemoglobin Analysis System (Bio-Rad Diagnostics, Hercules California) utilizing the principles of boronate affinity HPLC.

### Determination of PEPCK and G6Pase gene expression in liver of db/db mice by real time PCR

Total RNA in mouse liver was isolated by the TRIZOL^® ^method [39]. All RNA samples were DNased using the DNA-*free*™ kit (Ambion, Austin, TX) according to the manufacturer's protocol. The samples were then converted to cDNA using the High Capacity cDNA Archive Kit (Applied Biosystems, Foster City, CA) according to protocol. PCR results were generated using the 5' nuclease assay (TaqMan) [40] and the ABI 7900 Sequence Detection System (Applied Biosystems, Foster City, CA). Primers and probe for PEPCK are: Forward-TTGAACCTGAAAGGCCTGGG; Reverse-AAGGGAGGTCGGTGTTGACC; Probe-CGTCAACGTGGAGGAGCTGTTTGGGAT. Primers and probe for G6Pase are: Forward-CTGCATTGTGGCTTCCTTGG; Reverse-ATGCAAAGGGAACTGTTGCG; Probe-GCCCCCATCCCAGGTTGAGTTGATCTT. The primers and probe for Cyclophilin are: Forward-GGCCGATGACGAGCCC; Reverse-TGTCTTTGGAACTTTGTCTGCAA; Probe-TGGGCCGCGTCTCCTTCGA. The PCR cycling conditions were 95°C for 10 minutes, and 40 cycles of 95°C for 15 seconds and 60°C for 1 minute.

### Reagents

RU486 (Mifepristone, Cat. # M8046) and lipopolysaccharides (LPS, Cat. # L3755, Batch # 046K4068) were purchased from Sigma-Aldrich (St. Louis, MO).

### Statistical analysis

There were 5–9 mice for each data point. Data are presented as mean ± SEM. Differences between each pair of groups were analyzed with Student's t-test using JMP 6.0.0 software. A P value less than 0.05 was taken to be significant.

## Competing interests

The authors declare that they have no competing interests.

## Authors' contributions

BY is the principal investigator. YS, JAMcN, LGC, and PL participated in the experiments. PL, RPT, SAS, and GLP participated in the study design and manuscript preparation.
